# Participatory Causal Loop Diagrams Building for Supporting Decision‐Makers Integrating Flood Risk Management in an Urban Regeneration Process

**DOI:** 10.1029/2023EF003659

**Published:** 2024-01-29

**Authors:** Virginia R. Coletta, Alessandro Pagano, Irene Pluchinotta, Nici Zimmermann, Michael Davies, Adrian Butler, Umberto Fratino, Raffaele Giordano

**Affiliations:** ^1^ Department of Civil, Environmental, Land, Construction and Chemistry Polytechnic University of Bari Bari Italy; ^2^ Water Research Institute—National Research Council Bari Italy; ^3^ The Bartlett Faculty of the Built Environment Institute for Environmental Design and Engineering University College London London UK; ^4^ Department of Civil and Environmental Engineering Imperial College London London UK

**Keywords:** urban dynamics, flood risk management, causal loop diagrams, knowledge integration, Thamesmead

## Abstract

Several modeling tools commonly used for supporting flood risk assessment and management are highly effective in representing physical phenomena, but provide a rather limited understanding of the multiple implications that flood risk and flood risk reduction measures have on highly complex systems such as urban areas. In fact, most of the available modeling tools do not fully account for this complexity—and related uncertainty—which heavily affects the interconnections between urban systems evolution and flood risk, ultimately resulting in an ineffective flood risk management. The present research proposes an innovative methodological framework to support decision‐makers involved in an urban regeneration process at a planning/strategic level, accounting for the multi‐dimensional implications of flood risk and of different flood risk management strategies. The adopted approach is based on the use of System Thinking principles and participatory System Dynamics modeling techniques, and pursues an integration between scientific and stakeholder knowledge. Reference is made to one of the case studies of the CUSSH and CAMELLIA projects, namely Thamesmead (London), a formerly inhospitable marshland currently undergoing a process of urban regeneration, and perceived as being increasingly vulnerable to flooding. It represents an interesting opportunity for building a replicable modeling approach to integrate urban development dynamics with flood risk, ultimately supporting policy and decision‐makers in identifying mitigation/prevention measures and understanding how they could help achieve multi‐dimensional benefits (e.g., environmental, social and economic).

## Key Points

- Stakeholders' knowledge can provide relevant insights into the urban dynamics related to flooding
- Causal Loop Diagrams support better integrating flood risk management in evolving urban systems
- Behavior Over Time graphs support scenario analysis including the impact of prevention/mitigation measures

## 1 Introduction

Urban areas are experiencing an increasing level of flood risk mainly due to climatological and socio‐economic factors (Friedman, 2008; Kreibich et al., 2017). While the impacts of climate change on flood events—that is, variability of extreme events in frequency and intensity—are accounted for in the development and implementation of modeling approaches and tools for flood risk management, the dynamic nature of complex urban systems—which includes also socio‐economic elements such for example, population growth and distribution etc.—is largely ignored (Hemmati et al., 2020). This is a potential limit of the most widely used modeling tools and approaches for flood risk management in urban areas (Geltner & de Neufville, 2012; McInerney et al., 2012), which are mainly focused on a detailed assessment of bio‐physical aspects, without explicitly considering the relevant uncertainty associated to urban dynamics (Di Baldassarre et al., 2015; Perrone et al., 2020). Over the past century, stochastic and deterministic models for flood risk assessment had a relevant evolution, and started taking increasingly into account uncertainty and non‐stationarity in inputs, parameters, and structure using probability distributions, resulting in a “best‐guess output” (Hallegatte et al., 2013; Koutsoyiannis & Montanari, 2015, 2022; Montanari & Koutsoyiannis, 2014; Taleb, 2007). While several works deal with hydrological phenomena considering the concept of non‐stationarity (Salas & Obeysekera, 2014; Villarini et al., 2009), very few works (a) integrate the hydrological sub‐system with others (such as the social, economic, and environmental) (Wamsler et al., 2013) and (b) consider the influence of different elements—for example, built environment, population growth and distribution, infrastructure, green areas, etc.—on the impacts of extreme events in the urban system (Balch et al., 2020; Reed et al., 2022; Riddell et al., 2019). In general, a limited understanding of the complex interconnections between flood risk and urban dynamics could affect the effectiveness of policy and decision‐making processes related to the selection of the most effective actions for flood risk reduction (Kwakkel et al., 2010). Particularly, a poor understanding of the multiple benefits and impacts that different risk reduction measures might have is ultimately hampering a transition toward Nature Based Solutions (NBS, e.g., wetlands, swales, green roofs, etc.) which is recommended by the European Commission (European Commission, 2016) and supported by recent research (Albert et al., 2019; O’Keeffe et al., 2022; Palmer et al., 2015; Schanze, 2017).

To further understand how different factors interact within an urban system there is a growing need to benefit from different fields of knowledge (Hegger et al., 2012; Sanders et al., 2020). In particular, this requires the integration of scientific (i.e., provided by models and data) and expert/stakeholder knowledge during the various stages of model development (Kloprogge & Sluijs, 2006; Scrieciu et al., 2021; Voinov & Bousquet, 2010). This integration could be achieved using participatory modeling techniques, that is, building processes in which stakeholders are supported in the development and formalization of conceptual models (Carmona et al., 2013; Jordan et al., 2018; Voinov et al., 2016) and in the understanding of cross‐sectoral connections and implications (Ahmad & Simonovic, 2015; Moallemi et al., 2021; Pasquier et al., 2020). Nevertheless, the level of stakeholder involvement and the integration of their knowledge is still low in many modeling fields, including flood risk modeling (Scaini et al., 2021; Wehn et al., 2015), mainly due to time and funding constraints and the lack of trust that decision and policy‐makers have in participatory modeling approaches (Chilvers & Kearnes, 2016; Löschner et al., 2016).

Addressing urban system complexity calls for approaches that can analyze system structure and the dynamic relations among different urban components, described by non‐linear feedback loops (Bedinger et al., 2020; Xin et al., 2009). System Thinking—operationalized through System Dynamics (SD) modeling tools—help both considering the interactions between different variables and sub‐systems and dealing with dynamic problems and their combined effects (consequently highlighting potential trade‐offs and unintended consequences) (Giordano et al., 2020; Khan et al., 2009; Senge & Sterman, 1992; Simonovic, 2009). The value added of this approach has been widely discussed in recent works (see e.g., Egerer et al., 2021; Gastélum et al., 2010; Perrone et al., 2020; Stave, 2003; Tidwell et al., 2004; Videira et al., 2009) and mainly relates to: (a) the organization of scientific and stakeholder knowledge into a graphical structure that promotes a deeper understanding of problems (Rich et al., 2018); (b) the rigorous integration of different expertise and skills (Zomorodian et al., 2018); (c) the analysis of complex, non‐linear interactions among the different elements (qualitative/quantitative) including external drivers and pressures (Simonovic, 2009). Among the available SD modeling tools, Causal Loop Diagrams (CLDs) support the mapping of the system's feedback structure (Sterman, 2000) and have the potential to describe and analyze the complexity of a system, in a qualitative way, without dealing with mathematical computational load, incorporating scientific and stakeholder knowledge, and ultimately supporting decision‐making processes (e.g., Mashaly & Fernald, 2020; Phan et al., 2021; Senge, 1994; Susnik et al., 2012; Wyrwoll et al., 2018; Zomorodian et al., 2018). First, CLDs help in visualizing and identifying the underlying relationships and interactions within a system (such as urban systems), providing insights into how changes in one part may propagate and influence other elements (Meadows, 2008). Building a holistic perspective on the system help decision‐makers both avoiding the pitfall of oversimplifying the problem and address the root causes rather than just the symptoms (Richardson, 1997). Second, CLDs help in identifying leverage points in the system, that is, specific variables or elements that have a significant impact on the system’s behavior (Murphy, 2022). By focusing on these leverage points, decision‐makers can prioritize measures and identify and analyze effective strategies before implementation (Forrester, 1961). CLDs also support the iterative nature of the design process, as they can be easily modified and adapted as new information becomes available (Murphy & Jones, 2021). Moreover, due to their graphic nature, CLDs support discussion with/between stakeholders and provide a common visual language that can bridge the gap between experts and non‐technical stakeholders, facilitating effective communication and collaboration (Vennix, 1996). They can therefore be used to push participants to understand the complexity of connections in real systems (Coletta et al., 2021). These qualitative modeling tools can also support existing quantitative models used for assessing flood risk, particularly in the direction of identifying flood management strategies. Specifically, CLDs help prioritize key variables/interactions and measures to be included and tested in the quantitative model. This is especially beneficial when dealing with a high number of potential variables and complex interactions that can make data collection and analysis overwhelming (Rowell & Wormley, 1996). In addition, integrating insights from the qualitative modeling into quantitative models may lead to better model calibration and validation. Qualitative data might help refine model parameters and initial conditions, also in case of data scarcity, improving the accuracy and reliability of simulation results (Charles, 2003). This ultimately fosters greater stakeholder involvement and ownership of results, leading to more relevant and acceptable outcomes of the whole process (Perrone et al., 2020). Finally, considering that CLDs enable the identification of critical variables that significantly impact flood risk, by altering these variables and testing different scenarios within the CLDs, planners and decision‐makers can assess how different interventions might affect flood risk. This information can then be integrated into the quantitative models for more robust risk assessment and management (Kwakkel et al., 2015).

Within this framework, the present work uses System Thinking principles, implemented through a participatory SD modeling approach based on CLDs, for supporting the analysis of the complex interactions between flood risk assessment, risk reduction and urban dynamics. The present work aims to address the following research questions: (a) to what extent can flood risk and the impact of mitigation measures be jointly analyzed within a complex and dynamically evolving urban system (integrating e.g., physical, environmental, and socio‐economic aspects)? (b) to what extent can the proposed methodology, and in particular the use of Causal Loop Diagrams, help effectively integrating scientific and local knowledge for the purpose of analyzing potential future system pathways, thus supporting decision‐making processes?

The manuscript is organized as follows. Section 2 describes the different steps of the methodology, developed as part of the research projects CUSSH (Complex Urban Systems for Sustainability and Health) and CAMELLIA (Community Water Management for a Liveable London) focused on urban regeneration. Section 4 discusses the results obtained with specific reference to Thamesmead (London), an urban regeneration case study (introduced in Section 3) in which building resilience to flooding is considered a key issue for protecting both the community and the built environment. Section 5 shares the lessons learned from the implementation of the developed methodology.

## 2 Materials and Methods

### 2.1 Introduction to System Thinking

System Dynamics approach facilitates holistic understanding of complex dynamics systems, and strategic decision‐making (Ford, 1999; Richmond, 1993). This method operationalizes System Thinking, which seeks to understand interactions among the sub‐systems driving a system's overall behavior (Sterman, 2000).

Over the last decade, several studies have reviewed the various works that have applied a System Thinking approach to the management of water‐related issues (e.g., Mashaly & Fernald, 2020; Mirchi et al., 2012; Pejic Bach et al., 2020; Phan et al., 2021; Pluchinotta, Pagano, et al., 2021; Zarghami et al., 2018; Zomorodian et al., 2018) and some gaps in its application have emerged. In particular, the following were discussed in the most recent literature review by Phan et al. (2021): (a) few works have integrated scientific and stakeholder knowledge (Karimlou et al., 2020); (b) the ability of CLD to improve understanding of multiple interactions in complex water systems has not been fully exploited (Zare et al., 2019); (c) many studies have failed to validate the CLDs robustly, affecting their reliability especially when social, economic, and political sub‐systems, more difficult to predict than physically based sub‐systems, are included (Blair & Buytaert, 2016). From a methodological point of view, the main innovation proposed in this paper is related to the development of a qualitative model that: (a) is multi‐sectoral and allows all three components of risk to be considered (i.e., hazard, exposure, and vulnerability); (b) integrates scientific and stakeholder knowledge; (c) generates reliable assumptions about the dynamic evolution of urban systems in the event of flooding. Specifically, a CLD represents the structure of an interconnected system and creates a shared understanding of the system amongst members of a discussion group (Sterman, 2000). It consists of variables connected by causal links (arrows). Each arrow is assigned a polarity, either positive (+) or negative (−), which describes what would happen to the structure of the system if there were changes. A positive link means that if the cause increases/decreases, the effect increases/decreases; conversely, a negative link means that if the cause increases/decreases, the effect decreases/increases (Abebe et al., 2021; Coletta et al., 2021). Delays can also be added and are represented by a perpendicular double bar on the arrow; they give systems inertia and create oscillations and trade‐offs between the short‐and long‐term effects of policies (Sterman, 2000). In complex systems, the combination of positive and negative causal relationships in the CLD can form balancing (*B*) and/or reinforcing (*R*) feedback loops. The former generate balancing behavior that act as an equilibrator in a system; the latter contribute to the exponential behavior of a system (Sterman, 2000). As the behavior of complex systems arises from such relationships, analyzing the main feedback loops of the CLD allows the modeler to form hypotheses on the Behavior Over Time (BOT) of an urban flood system. This ultimately supports an understanding of what the main implications and potential impacts could be, avoiding undesirable future scenarios (e.g., Braun, 2002; Senge, 1994). The basic BOT in dynamic systems are: (a) exponential growth/decline, created by self‐reinforcing feedback loops; (b) goal seeking behavior, that arises from balancing feedback loops; (c) oscillation, that can occur if there are delays in at least one of the links in a balancing loop, causes the system to constantly move above and then below its goal (i.e., the desired state of the system) (Sterman, 2000). Interactions of these BOT may cause three more complex patterns of behavior, that is, S‐shaped Growth, S‐shaped Growth with Overshoot, Overshoot and Collapse (e.g., see Mirchi et al., 2012 for further details). For best practice of building/using CLD see Haraldsson (2004).

### 2.2 Overview of the Proposed Approach

This section provides a detailed description of the methodological framework adopted, based on a multi‐step process of knowledge gathering and on the System Thinking principles. Table 1 shows the different activities with their objectives, the tools/methods adopted, and the expected results. The common ground for the different phases of the modeling approach is the active participation of stakeholders, supported by different methods described in the following sections. The process includes both a qualitative and quantitative modeling phase. The present work focus on the former (Tasks 1–5) but also includes some information on the latter (summarized in Task 6) at the end of this Section.

**Table 1:** The Adopted Multi‐Step Process

No.	Tasks	Aims	Tools/Methods	Outcomes
1	Literature review and baseline analysis for preliminary Causal Loop Diagram (CLD) building	To build a preliminary CLD, based on the scientific knowledge and background information on the study area	Literature review on urban flooding Gathering information about the study area, for example, from reports, existing models, etc.	A preliminary CLD on the study area, based on the scientific knowledge, focused on urban flood risk
2	Interviews with stakeholders for preliminary CLD improvement	To collect and structure stakeholder knowledge for improving the key cause‐effect chains of the preliminary CLD	Semi‐structured interviews with stakeholders and email exchange Analysis of semi‐structured interviews Integration of scientific and stakeholder knowledge	A CLD on urban flood risk which integrates scientific and stakeholder knowledge
3	CLD causal structure validation	To validate general structure and key CLD connections	Collective model testing and participatory exercises	Final structure of CLD
4	Behavior Over Time (BOT) graphs construction with stakeholders	To collect stakeholder perception on the dynamic evolution of some key variables of the system	BOT graphs construction	Graphs on the dynamic evolution of the system based on stakeholder perception
5	CLD integration based on stakeholder‐built BOT graphs	To analyze the main dynamics and impacts of flood in the CLD To integrate BOT graphs results into final CLD	BOT graphs and key CLD feedback loops analysis	Formulation of hypotheses on urban system dynamics and flood risk management policies
6	Stock and Flow (SF) model building and scenario analysis	To develop a simulation model that integrates urban dynamics and flood risk To select the most suitable adaptation strategy	Data collection Participatory exercises Sensitivity analysis	A SF model on urban flood risk integrating scientific and stakeholder knowledge and recommendations for implementing a suitable adaptation strategy

*Note*. The activities with their objectives, the tools/methods adopted, and the expected results are shown.

#### 2.2.1 Task 1: Preliminary Causal Loop Diagram Building

The aim of the first modeling activity (Task 1) was to build, based on both scientific knowledge on the main physical phenomena and specific information on the case study, a preliminary CLD to explicitly relate the issue of flooding to the main urban dynamics of the area. Whereas often a CLD can be directly co‐developed with the stakeholders (see e.g., Inam et al., 2015; Perrone et al., 2020), existing scientific papers and models were used in the present work to develop a model draft which was subsequently augmented with stakeholder knowledge. This choice helped significantly expanding the level of detail of the model. For this purpose, an in‐depth analysis of (a) the main variables involved in hydraulic flood models existing in the literature, with focus on urban areas and (b) background information on the study area, for example, from reports, existing models, etc., was necessary. Scientific electronic databases (Scopus, Web of Sciences, and Google Scholar) were used to identify original research and academic papers on flood risk in urban contexts. Some keywords, such as “flooding,” “flood risk,” “cities,” “urban dynamics,” were combined to select relevant articles. Regarding the specificities of the case study, Google searches and email exchanges with involved stakeholders proved invaluable. When formal equations were identified within the selected scientific papers and/or existing models, their transformation into CLD cause‐effect relationships was done under the assumption that: (a) the terms of the equations represent variables, for example, *A* and *B*; (b) the correlation between the variables depends on whether variable *B* modifies variable *A*; (c) if the variation of *A* with respect to *B* is greater than zero, the polarity of the connection is positive, whereas if the variation of *A* with respect to *B* is less than zero, the polarity of the connection is negative (Sterman, 2000). For example, in the equation that links runoff coefficient (*C*) to river peak discharge (Qp), if *C* increases (decreases), then Qp increases (decreases). This means that the variation of Qp with respect to *C* is greater than zero. This relationship can be represented in the CLD by an arrow, with positive polarity, that starts from the variable “surface runoff” and arrives at the variable “river peak discharge.”

#### 2.2.2 Task 2 and 3: Stakeholder Involvement and Causal Loop Diagram Causal Structure Validation

Task 2 mainly aims at gathering the stakeholder perception about flood risk and past flooding events in the chosen area and at integrating it into the CLD. Semi‐structured interviews with stakeholders were conducted. Stakeholder interviews were used to elicit the perception of the system boundaries and individual problem framing, and provide useful insights to add, modify, or improve the cause‐effect relationships of the diagram (Inam et al., 2015; Kotir et al., 2016; Pluchinotta, Salvia, & Zimmermann, 2021). With the support of an agenda, each question was associated with an objective. Specifically, the first question helped the modeler understand why it is important to investigate flooding in the area. The interviewer asked the respondents whether, to their knowledge, flooding events had occurred in the past. If they had occurred, subsequent questions had the following objectives: (a) collecting information on past flooding events; (b) understanding what type of flooding the area is most susceptible to; (c) investigating damage due to flooding; (d) investigating the effectiveness of individual and collective flood risk prevention measures in the area; (e) investigating what post‐intervention measures had been taken. If, however, the interviewee did not recall past flood events, the objectives of the following questions were: (a) understanding whether the non‐occurrence of flood depended on the exposure of the system to risk, for example, the absence of heavy or long‐lasting rainfall, or on the effectiveness of risk mitigation measures, for example, drainage systems; (b) understanding why it could be important to investigate flooding. The interview structure can be found in Table S1 of the Supporting Information S1. The responses given by the stakeholders were then translated into variables and the causal interconnections into links in the CLD. For this purpose, the stakeholders' responses were analyzed based on the identification and use of specific categories: (a) cause variables; (b) effect variables; and (c) causal relationship type, that is, positive or negative polarity (Eker & Zimmermann, 2016; Kim & Andersen, 2012). Table 2 below provides a couple of examples of this process. In case of divergences of problem frames, they were aligned promoting discussion between stakeholders during a participatory workshop aiming at validating the CLD causal structure (Task 3).

**Table 2:** Examples of the Analysis of the Semi‐Structured Interviews With Stakeholders

Quotes from the interviews	Cause variable	Effect variable	Relationship type
“With improved defenses the risk of flooding from river is residual”	River defenses effectiveness	River flood	Negative
“Part of the road floods almost to the extent that the whole road is underwater. It affects people's movements and lives in general”	Infrastructure damage	Productive activities operation Economic losses Residents' health	Negative Positive Negative

As stated by Mirchi et al. (2012), in participatory System Dynamics modeling, the validation can be done with the involvement of a range of experts and stakeholders during different phases of the modeling process. For this reason, several works have validated their System Dynamics models on water problems through the consultation of stakeholders (see e.g., Bertone et al., 2019; Pagano et al., 2019; Sahin et al., 2016; Susnik et al., 2012), which is particularly relevant when expert knowledge is used for model building. In this work, a workshop was organized, and stakeholders were asked to provide comments on both specific parts of the model and the whole CLD structure. Adopting the semi‐structured interview style, a facilitator presented the model to stakeholders by the division of the variables into thematic clusters and encouraged them to validate the uncertain connections. Specifically, relationships between variables on which the literature was insufficient or for which stakeholders expressed different views in Task 2, were discussed. This provided the final architecture of the CLD, which thus integrates scientific and stakeholder knowledge. It is important to clarify that the CLD does not represent a unique and definitive view of the analyzed system, but a description based on the available knowledge, which can be subject to updates and revisions.

#### 2.2.3 Task 4: Behavior Over Time Graphs Construction

Task 4 represented an additional step of knowledge building through stakeholder engagement. While the previous activities (Task 2 and 3, Section 2.2.2) concerned the collection of the stakeholders' understanding of the cause‐effect chains affecting flood risk, in this task their perception of the dynamic evolution of the urban system and flood risk was collected. A workshop was organized, and stakeholders drew and described BOT graphs of key variables under different conditions. These variables were the main elements of the urban system affected by flooding, for which data are limited or unavailable. Although in the literature the construction and analysis of BOT graphs mainly support the problem structuring phase before the CLD construction with stakeholders (see e.g., Cavana & Maani, 2004; Elias, 2012), in this work this exercise was performed afterward as the preliminary CLD was built based on scientific knowledge. For this reason, the BOT graphs represented a further step of stakeholder knowledge elicitation, necessary for building the final CLD and therefore for the formulation of hypotheses about the dynamics of the urban system related to flooding. Indeed, BOT graphs have the potential to provide insights and inform future modeling and data collection priorities (Calancie et al., 2018). In addition, integrating a multiplicity of perspectives may help practitioners understand the consequences (intended or unintended) of potential interventions and therefore formulate more realistic assumptions about the dynamics of the urban system (Hovmand et al., 2013). After presenting the basic BOT in dynamic systems and giving an example of how to draw a BOT, a facilitator organized stakeholders into groups based on their expertise. In each group the facilitator asked stakeholders to draw each variable's BOT under three different future conditions: (a) desired future, that is, the evolution of the variable as the stakeholders would prefer; (b) most likely future, that is, the evolution that the variable is expected to have; (c) feared future, that is, the evolution of the variable that stakeholders do not want. Such graphs were built highlighting—if possible—specific values and thresholds. The groups were then brought together, and each group briefly presented at least one BOT graph. Variables that were particularly difficult to quantify, for example, variables that are intangible and related to attributes of human behavior, were assigned to all groups. When the graphs drawn by the different groups on the same variables did not show relevant differences, the graph that contained most information and a higher‐level of detail was chosen. In case of large differences, the various interpretations were evaluated by all stakeholders to reach a consensus. At the end of the workshop an evaluation form of the activity was submitted to the stakeholders to help the facilitator improve future participatory modeling activities.

#### 2.2.4 Task 5: Causal Loop Diagram Integration

To formulate hypotheses on both urban system dynamics and the implementation of flood related policies, Task 5 was based on the aggregation of the knowledge collected in the previous activities. Specifically, the CLD—and more precisely the information provided by the feedback loops—was integrated with the stakeholder‐built BOT graphs.

Although one of the advantages of CLD is that the essential components and interactions in a system can be represented with simplifications (Haraldsson, 2004), Richardson (1997) and Lane (2008) demonstrated with some examples the impossibility of rigorously inferring dynamic behavior from non‐formal models, such as CLD. Indeed, considering that the behavior depends upon rate‐to‐level links, hidden loops, and net rates, that are unspecified in the CLD, traditional definitions in terms of behavior (based on CLD polarities) affect the fairness of dynamic behavior inferred from feedback loops. In addition, Schaffernicht (2010) stated that CLDs: (a) draw attention on “events,” that is, a discrete change in one of the aspects of behavior, rather than on the behavior itself; (b) show system structure only, leaving the behavioral aspects to the modeler; (c) do not represent aspects of the structure that help the modeler notice traps of behavioral inferences. These problems are exacerbated in the case of a multi‐loop system, such as an urban one. To make the assumptions about the dynamic behavior of the system deduced from the feedback loops of the CLD as reliable as possible, the potential of the CLD was expanded through its integration with the BOT graphs built by stakeholders.

#### 2.2.5 Potential Application of the Qualitative Modeling Framework

The insights from the qualitative modeling phase described in this study will support a better understanding of the system, which will help: (a) the construction of the quantitative System Dynamics model (i.e., the Stock and Flow (SF) model), that will also integrate the results of sectoral models (including flood risk assessment models); (b) the analysis of the multi‐dimensional benefits associated with the implementation of the selected measures, and (c) the simulation of different future scenarios of intervention. From a practical point of view, the key variables and causal relationships of the CLD will be translated into SF model sets (see Sterman, 2000). The BOT graphs built with the stakeholders will be employed for both the identification of the initial value of key variables of the urban system for which there is a lack of data/information, or which are difficult to quantify (such as environmental and socio‐economic benefits), and for the validation of the model structure and potential intervention scenarios. Hence, the results of the qualitative modeling will be a fundamental starting point for identifying the most suitable strategy for the analyzed system.

## 3 The Thamesmead Case Study From the Flood Risk Analysis Perspective

**Figure 1:**
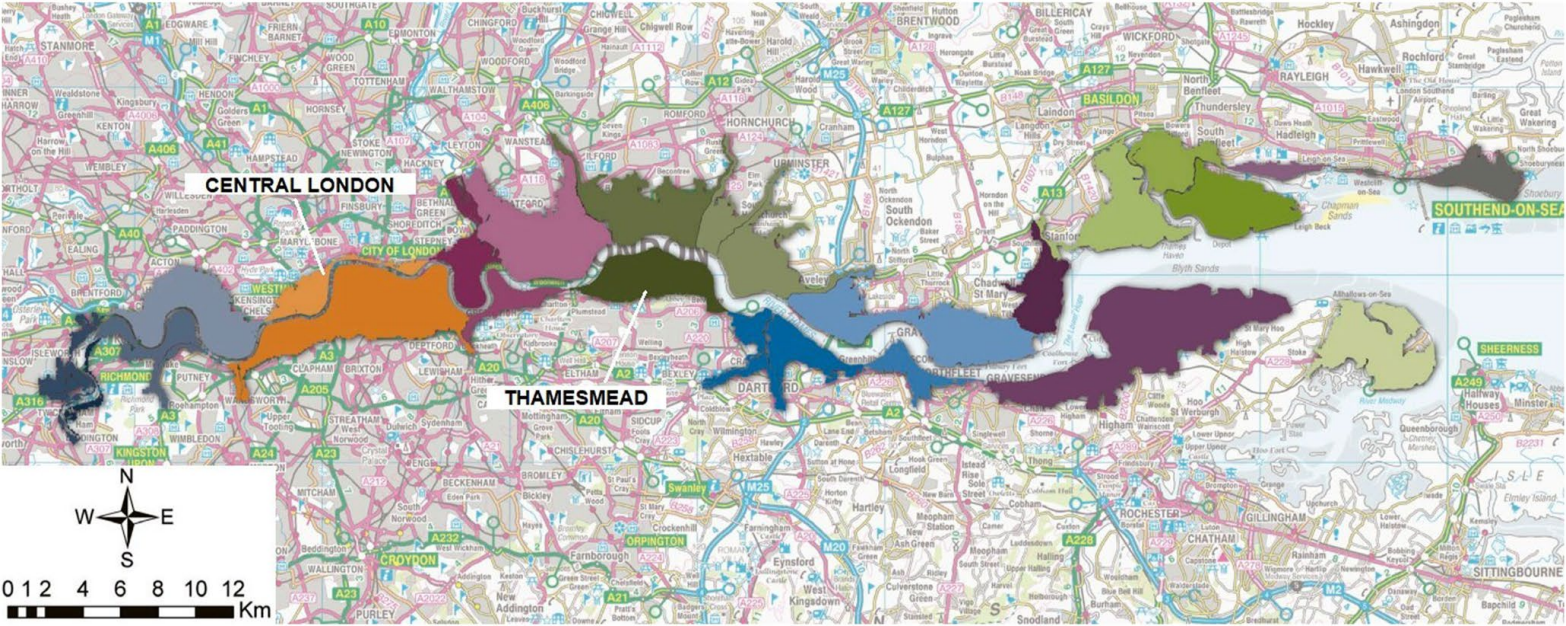
Overview of the study area in relation to central London (adapted from EA (2012)). The colored areas are the eight Thames Estuary 2100 flood risk action zones.

The proposed methodology has been applied to the Thamesmead (TM) case study, a former inhospitable marshland in south‐east London, drained in the 1960s when the Greater London Council bought it with the aim of transforming the land into an attractive residential area (Markowitz, 2017). A new regeneration plan renewed the interest in flood risk in the area (Peabody, 2019). The area (Figure 1) is located between the London Borough of Bexley and the Royal Borough of Greenwich. It is bordered by Woolwich to the southwest, Abbey Wood to the south, Belvedere and Erith to the southeast, and the tidal River Thames to the north.

The information in this section was mainly gathered from the Thames Estuary 2100 Plan (Environment Agency, 2012), the Living in the Landscape Framework (Peabody, 2021), the Charlton to Bexley Riverside Integrated Water Management Strategy (AECOM, 2017), and from semi‐structured interviews or personal correspondence with stakeholders. In this regard, additional details are included in Table S2 of the Supporting Information S1.

During other participatory modeling activities within the CUSSH and CAMELLIA projects focused on the definition of actions for increasing the quality of Built/Blue‐Green (BG) environment in Thamesmead (see Pluchinotta, Salvia, & Zimmermann, 2021 for further details) building resilience to flooding was mentioned as a key issue for protecting both the local community and the built environment in the area by some stakeholders. The area, in which there are 21 schools, six care homes, and over 100 electricity sub stations, is vulnerable to four types of flooding mechanisms: tidal, fluvial, pluvial, and groundwater flood. Considering that TM is a large area of reclaimed land and the topography is low‐lying and very flat, flood depths in an extreme surge tide event could breach the local defenses (i.e., River Wall and two sections of embankment). Furthermore, the embankment of some rivers and the increase of the area's imperviousness make the risk of pluvial flooding particularly serious. In addition, where the capacity of the drainage system is low, the flooding risk is high. The Thames Water Southern Outfall Sewer runs through the area and the drainage system is dominated by a system of lakes and canals, which is London's largest Sustainable urban Drainage System (SuDS), and the Erith Marshes system of ditches and dikes. Lastly, as the base geology is largely permeable, the area is considered to have potential for groundwater flooding.

Based on all these considerations, the integration of flood risk management into urban regeneration dynamics has been explored. Furthermore, the COVID‐19 pandemic (and related restrictions) forced the research team to reorganize the activities with limits on meetings location/duration and on the number of stakeholders involved in the process. For these reasons, a large stakeholder engagement for this specific model was not possible and meetings and workshop were held only online with an interest group. Therefore, for this model, a relevant group of local technical stakeholders (flood experts, representatives of relevant organizations or institutions as reported in Table S2 of the Supporting Information S1) was identified to provide expert knowledge to better understand the potential interactions between the urban development and flooding in the area. Particularly, during the first round of interviews, each interviewee that mentioned the issue of flooding was asked to suggest the involvement of another relevant stakeholder (in terms of role and expertise) to be invited in the following meetings (the snowballing approach described in Reed et al. (2009) was used). Considering the context and overall constraints described above, one of the aims of this modeling process was also to build a long‐lasting engagement and relationship with/between stakeholders with interest in flooding, to overcome their way of working in silos. Moreover, since the modeling focus, boundaries and objectives were defined with the stakeholders as part of the two research projects (CUSSH and CAMELLIA), the participatory modeling process presented in this paper could be defined as “expert‐based” (Barreteau et al., 2010).

## 4 Results

### 4.1 Thamesmead Flood Causal Loop Diagram Construction

**Figure 2:**
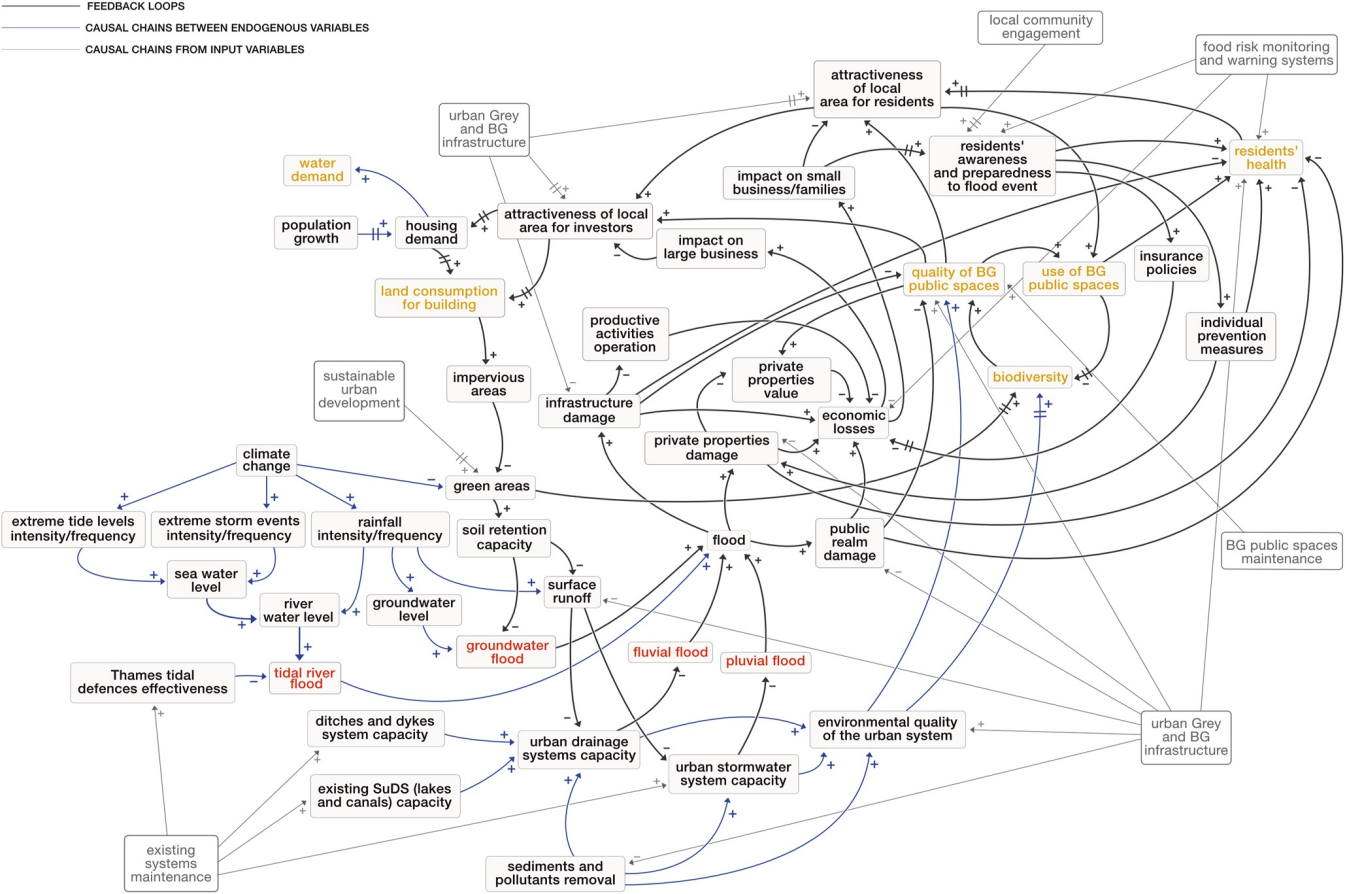
Thamesmead flood CLD. Variables in red are the flooding mechanisms; variables in orange define the main elements currently explored in CUSSH and CAMELLIA projects; variables in gray identify the main measures. The links between variables within feedback loops (endogenous variables) are in black while simple causal relationships are in blue and gray.

The final version of the CLD related to the TM study area is shown in Figure 2. The variables in red identify the four main types of flooding mechanisms (i.e., tidal river flood, groundwater flood, fluvial flood, and pluvial flood) to which the area is vulnerable. The variables in orange identify the main issues/elements that are currently explored within the CUSSH and CAMELLIA projects and that represent a “basis” for the developed model (see Davies et al., 2021); those in gray define the main measures/actions that, based on literature, stakeholder knowledge, and the ongoing projects, could be implemented in the area. The links between variables within feedback loops (endogenous variables) are black to distinguish them from the simple causal relationships (in blue and gray). A full list of the variables used in the CLD, along with a description, is provided in Table S3 of the Supporting Information S1.

**Figure 3:**
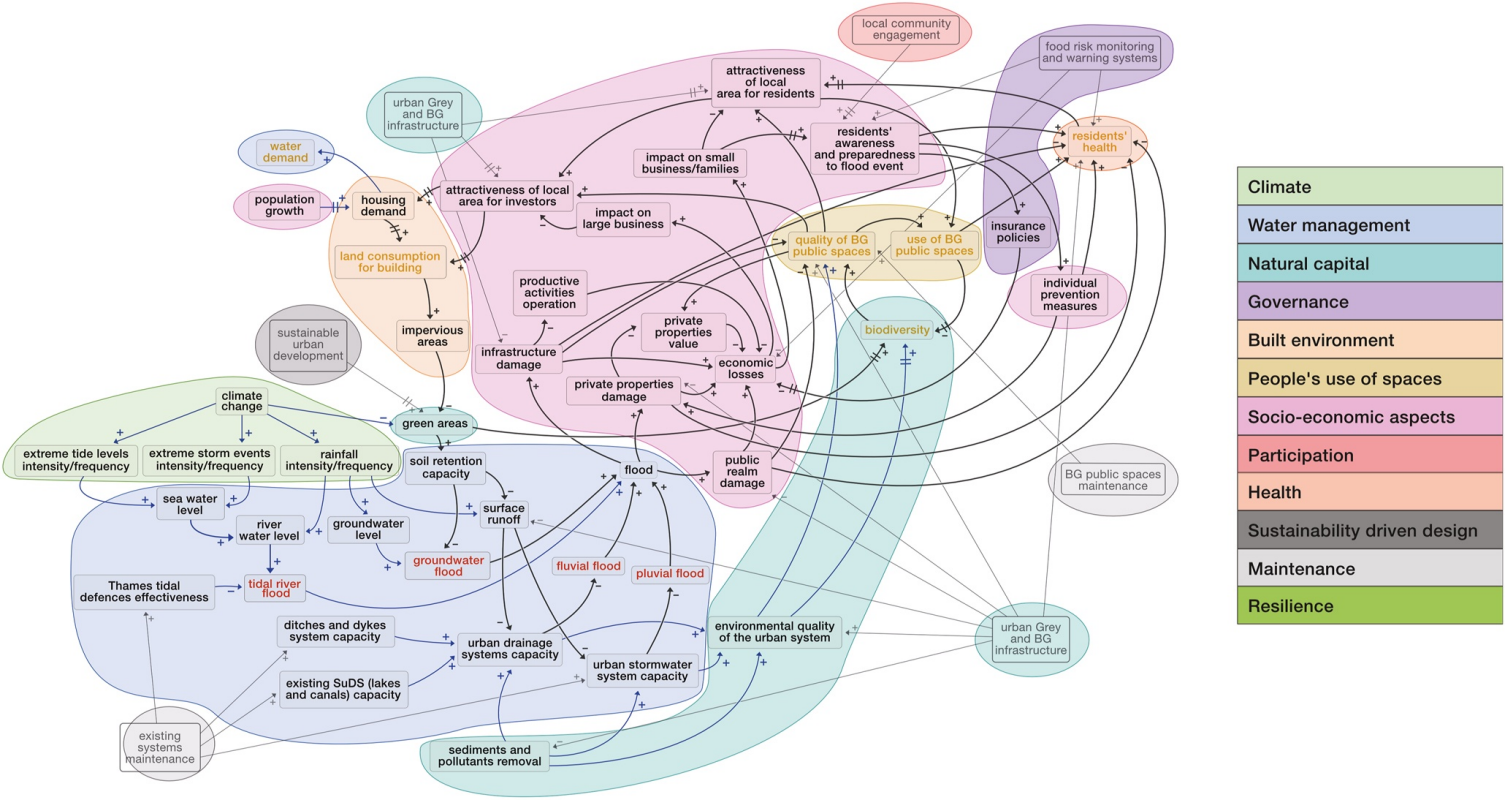
Thamesmead flood CLD with thematic clusters highlighted.

The CLD in Figure 2 was obtained using the Vensim® software, following Tasks 1, 2, and 3 described in Section 2.2. The information needed to build the preliminary TM urban flood risk CLD (Task 1, Section 2.2.1) has been taken from (a) literature review on hydraulic flood models variables, for example, soil retention capacity and surface runoff, and the topics of flood risk and its effects on urban systems in general; (b) other TM CLDs already developed through three previous stakeholders workshops (between January and July 2020) on the quality of the built environment and BG spaces for other ongoing modeling activities within the CUSSH and CAMELLIA projects (see Pluchinotta, Salvia, & Zimmermann, 2021 for further details); (c) existing water management reports through London and the Thames estuary obtained from involved stakeholders (see Table S2 in Supporting Information S1). As far as the improved CLD version which includes stakeholder knowledge (Task 2, Section 2.2.2) is concerned, it was obtained from the analysis of both four rounds of semi‐structured interviews of approximately 1 hr duration (see Table S1 in Supporting Information S1) and the review of past flooding events in the area with experts. The validation of both some key connections and the general structure of the improved CLD with stakeholders during an online workshop (approximate duration 1 hr) held on 9 September 2021 allowed producing the final version of the CLD structure (Task 3, Section 2.2.2). CLD validation was based on the use of semi‐structured interviews. Full details on the workshop agenda used for the TM CLD causal structure validation and on the stakeholders involved in the workshop are in Tables S4 and S5 of the Supporting Information S1 respectively. With the aim of identifying and labeling features in the variable set and being also consistent with the analysis of the other CLDs on the quality of built/BG environment already developed for the case study, the TM CLD variables were coded into first order thematic clusters identified by the CUSSH/CAMELLIA team (see Pluchinotta, Salvia, & Zimmermann, 2021 for further details). Four coders carried out the attribution of thematic clusters to the CLD flood variables. Fifteen variables were attributed to the “water management” sector; 13 variables were included in the “socio‐economic aspects” cluster. In the “natural capital,” “climate,” and “built environment” sectors, five, four, and three variables have been allocated respectively. The remaining variables of the flood CLD were instead distributed, for a maximum number of two variables per sector, between the clusters “people’s use of spaces,” “health,” “participation,” “maintenance,” “governance,” and “sustainability driven design.” Figure 3 shows the TM flood CLD with the first order thematic clusters highlighted.

The second part of the online workshop held on 9 September 2021 (approximate duration of 1 hr) and oriented to the analysis of the BOT graphs of key variables of the system (Task 4, Section 2.2.3). Full details on the workshop agenda used for the BOT graphs construction are included in Section 4 of the Table S4 in Supporting Information S1. Using Jamboard, the digital interactive whiteboard developed by Google, the stakeholders were asked to represent and describe, with the help of facilitators, the behavior over time of the variables “infrastructure/public realm damage” and “private properties damage” due to flooding, “quality of BG public spaces,” “attractiveness of local area,” and “residents' health” under the three different conditions introduced in Section 2.2.3 of the methodological framework (i.e., desired future, most likely future, and feared future). The variables listed above were chosen for a twofold reason. First, they represent some of the objectives set by Peabody’s ambitious Plan for regeneration in TM (namely, minimizing flood damage, achieving an attractive neighborhood and high‐quality BG public spaces, and improving the well‐being of residents). Second, the possibility of finding data that describe them over time is limited. The seven stakeholders who participated in the workshop were divided into two groups in relation to their expertise and interests. The first group with four stakeholders was responsible for representing the variables “infrastructure/public real damage” and “private properties damage”; the second group worked on the graphs of the variables “attractiveness of local area” and “residents' health.” The variable “quality of BG public spaces” was assigned to both groups, because it was considered particularly difficult to represent due to the absolute lack of data in the literature. The time horizon considered in the graphs was from 2010 until 2050, that is, the end of the regeneration Plan.

### 4.2 Causal Loop Diagram Integration Based on Behavior Over Time Graphs

In this section, the mechanisms of the CLD which have the same variables as the BOT graphs are analyzed and enriched to hypothesize the dynamic behavior of the variables. Task 5 of the methodological framework (Section 2.2.4) focused on formulating hypotheses on both urban system dynamics and the implementation of policies in the context of flood risk. Within the flood CLD, through the application of function “loops” in Vensim® software, 396 feedback loops directly involving the variable “flood” have been identified. Specifically, 132 involve “pluvial flood,” “groundwater flood,” and “fluvial flood.” The loops that are produced are mainly balancing loops. No feedback loops involve the variable “tidal river flood.” The loops chosen for the analysis and integration with BOT graphs are those that contain a greater number of variables identified as important by the stakeholders in previous activities carried out within the CUSSH and CAMELLIA projects (“land consumption for building,” “biodiversity,” “use of BG public spaces,” “economic losses,” “impact on small business/families”). The variables involved in each feedback loop, the related dynamics activated within the system, and the behavior mode are included in Table S6 of the Supporting Information S1. For the sake of brevity, only the most relevant CLD‐BOT graphs' integrations are presented below. These involve the feedback loops B1 and B2, whose dynamics mainly relate to the variables “infrastructure damage” and “public realm damage,” and the B4 and B5, whose dynamics are related to “attractiveness of the local area” and “quality of BG public spaces” respectively.

#### 4.2.1 Infrastructure and Public Realm Damage

**Figure 4:**
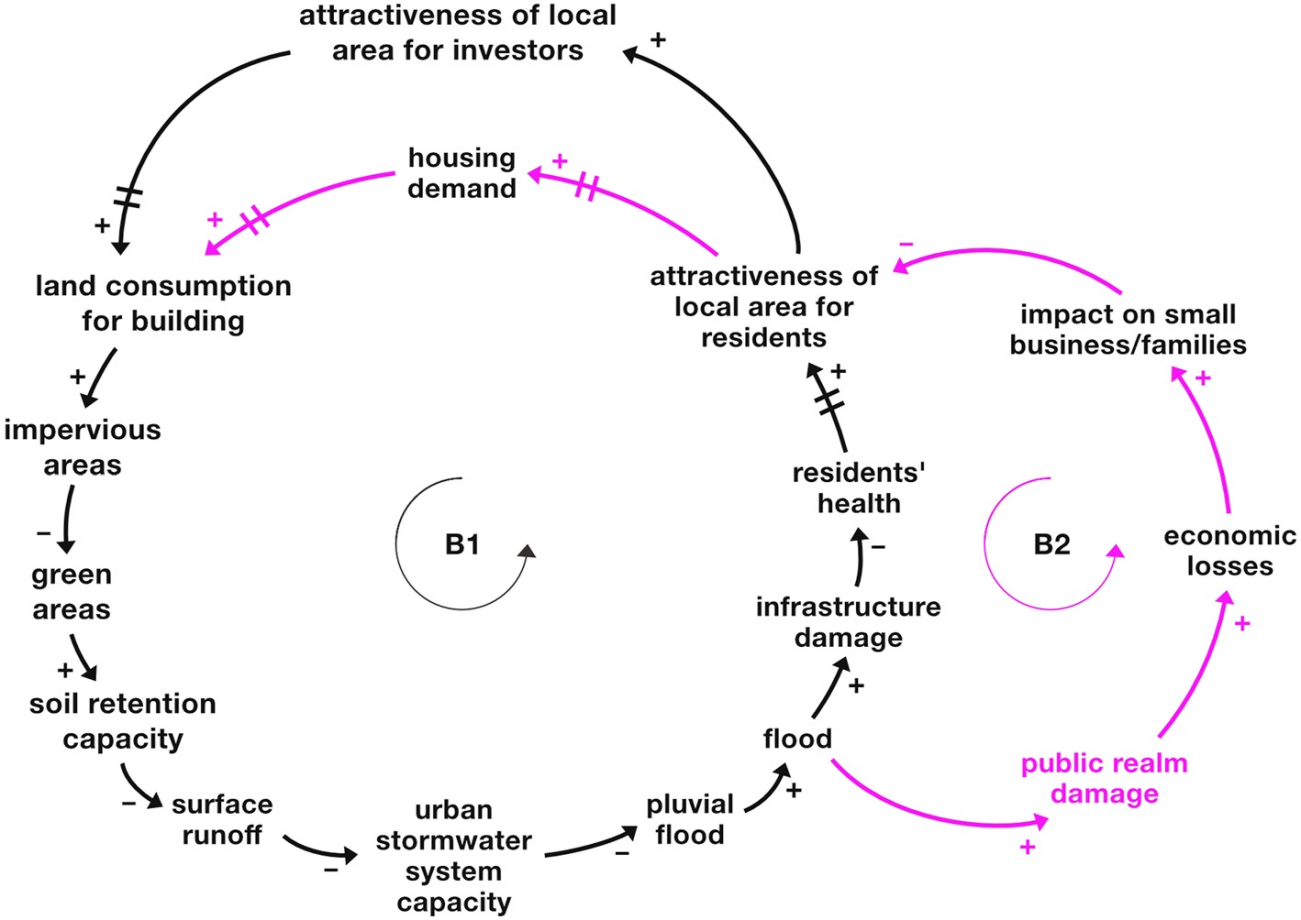
Infrastructure and public realm damage feedback loops (respectively B1 and B2). Loop B1 is the one in black, while loop B2 is the one in pink. The variables “land consumption for building,” “impervious areas,” “green areas,” “soil retention capacity,” “surface runoff,” “urban stormwater system capacity,” and “pluvial flood” are in common between the two loops. Changing times (delays) are represented by bars on the arrows.

First, two balancing loops with time delays involving the “infrastructure damage” (B1) as well as the “public realm damage” (B2) are isolated and shown in Figure 4. The minimization of both classes of damage is a key objective for the area.

Specifically, the black balancing loop B1 shows that an increase of “flood” may lead to an increase of “infrastructure damage” with a consequent reduction of “residents' health” and the attractiveness of the area. Considering the balancing loop B2 (pink), if “flood” increases, the “public realm damage” and “economic losses” increase, reducing the attractiveness of the area. In both loops a reduction of the attractiveness of the area might lead to a decrease of “land consumption for building,” resulting in an increase of “soil retention capacity” and a reduction of flood risk. These are two balancing feedback loops with delays that might create oscillating behavior in the system in relation to the achievement of the established objective, that is, the minimization of the “infrastructure/public real damage.” This means that flood damage to infrastructure and the public realm may either increase or decrease in different conditions. Both loops are closely interconnected due to shared variables (“flood,” “attractiveness of local area for residents,” “land consumption for building,” “impervious areas,” “green areas,” “soil retention capacity,” “surface runoff,” “urban stormwater system capacity,” and “pluvial flood”). Thus, if one of the two types of damage is reduced, the other one could be reduced as well.

**Figure 5:**
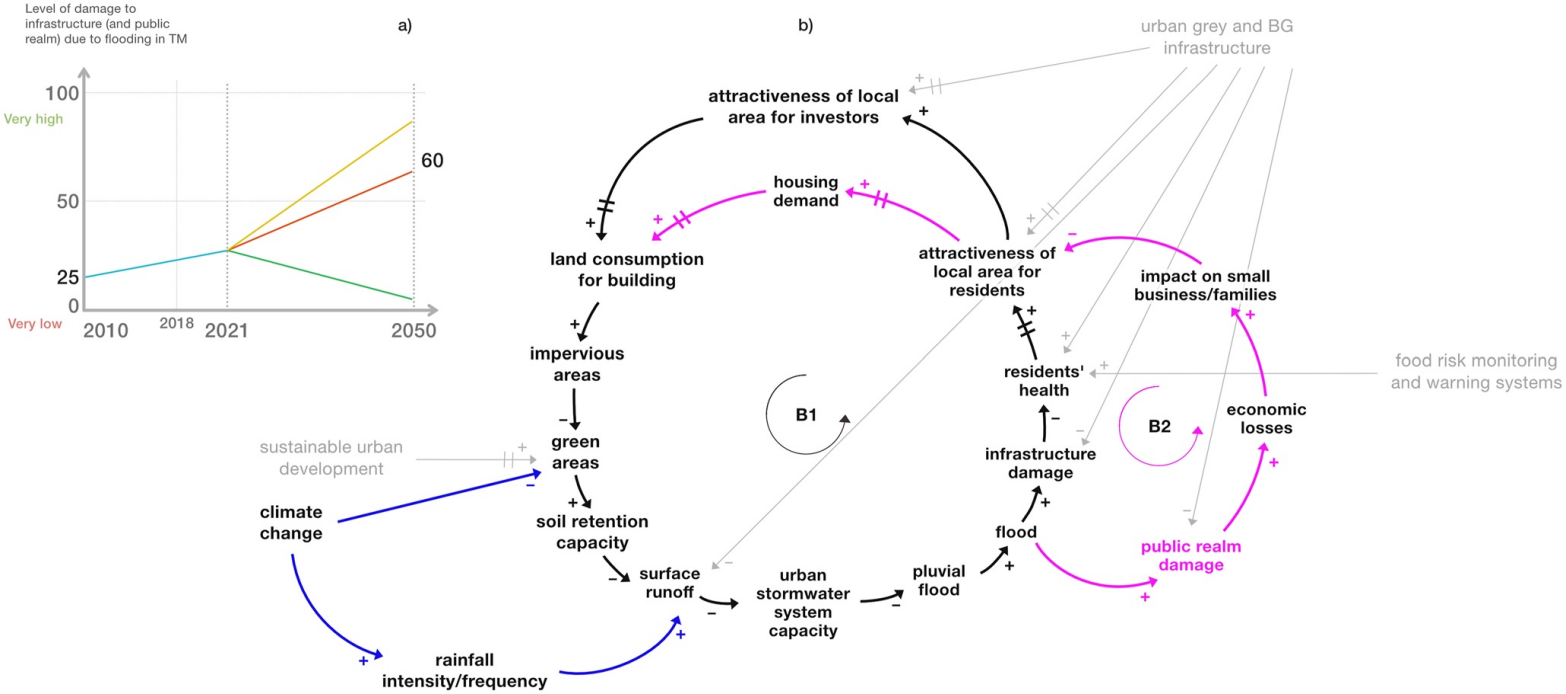
(a) BOT graph of the variables “infrastructure/public realm damage” created by the stakeholders during a participatory workshop. The blue line represents the past behavior of the variable, the yellow, red, and green ones respectively the feared, most likely, and desired future. (b) Infrastructure and public realm damage feedback loops (respectively B1 and B2). Loop B1 is the one in black, while loop B2 is the one in pink. The variables “land consumption for building,” “impervious areas,” “green areas,” “soil retention capacity,” “surface runoff,” “urban stormwater system capacity,” and “pluvial flood” are in common between the two loops. The variables in gray are measures/actions; simple causal relationships between variables outside the feedback loops (exogenous variables) are in blue. Changing times (delays) are represented by bars on the arrows.

Figure 5 below shows the feared future (yellow line) and the most likely future (red line) of the variables “infrastructure damage” and “public realm damage” as perceived (and drawn) by stakeholders; according to them, both trends may increase over time due to the impacts of climate change.

Considering the effect of the variable “climate change” on the loops (Figure 5b), it can be observed that a large increase of the variable may lead to a significant decrease of “green areas” and a large increase of “surface runoff” with a consequent increase of “infrastructure damage” and “public realm damage” and thus a linear upward trend of the variables (instead of oscillatory as would result from the analysis of the loops alone in Figure 4). Depending on the severity of the effect of “climate change” on “green areas” and “surface runoff,” the feared and most likely future may be obtained. According to the stakeholders, by activating flood risk mitigation/prevention measures in the system, the variables “infrastructure damage” and “public realm damage” may behave similarly to the desired future (green line, Figure 5a), which is linearly decreasing. In fact, adding some interventions in the loops simultaneously (see Figure 5b) may generate the desired dynamics and thus move from an oscillatory to a linear trend of damage minimization. For example, the introduction of “urban gray and BG infrastructure” may allow the rebalancing of the system thanks to an effect on the “infrastructure damage” and “public realm damage” variables. In the long term, further corrective measures, such as “sustainable urban development,” should be activated to ensure that the system does not move away from the target of damage minimization (and therefore from the desired future). Indeed, short‐term damage management (e.g., through the introduction of “urban gray and BG infrastructure”) may lead to an increase of the attractiveness of the area and of “land consumption for building,” which, if not effectively controlled, risks reducing “soil retention capacity,” which is increasingly exacerbated by “climate change,” and once again unbalancing the system.

#### 4.2.2 Attractiveness of Local Area and Quality of Blue and Green Public Spaces

**Figure 6:**
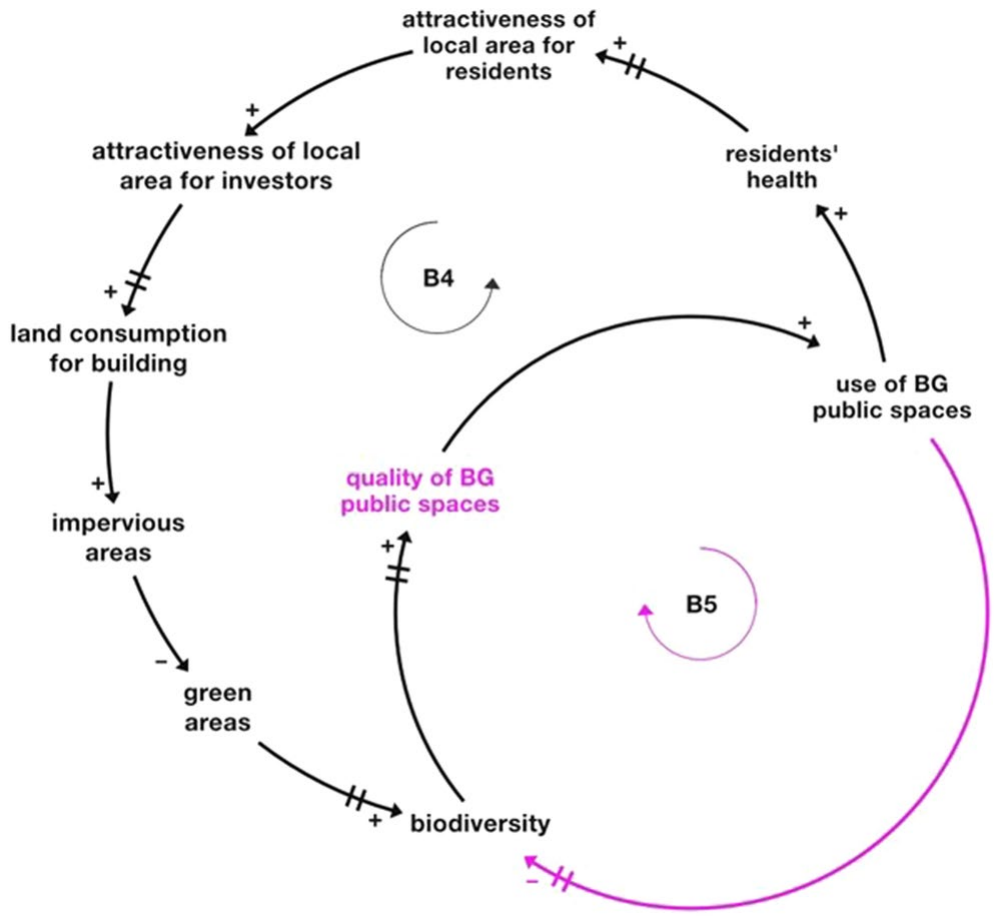
Attractiveness of local area and quality of Blue‐Green (BG) public spaces feedback loops (respectively B4 and B5). Loop B4 is the one in black, while loop B5 is the one in pink. The variables “biodiversity,” “quality of BG public spaces,” and “use of BG public spaces” are in common between the two loops. Changing times (delays) are represented by bars on the arrows.

Figure 6 includes two balancing feedback loops with time delays (B4 and B5) related to the “attractiveness of local area” and “quality of BG public spaces.” Key objectives for the area are the achievement of an attractive neighborhood and high‐quality BG public spaces.

The loops show how “attractiveness of local area” and “quality of BG public spaces” may increase or decrease as variables belonging to other thematic clusters, that is, built environment, natural capital, and space use, change. Focusing on the black balancing loop B4, if “land consumption for building” increases, “green areas” may decrease as well as “biodiversity,” leading, in the long run, to a reduction in the attractiveness of the area in general and consequently in the “land consumption for building.” The pink balancing loop B5 shows instead what happens to the system if “biodiversity” decreases or increases. If “biodiversity” decreases, the quality and use of BG public spaces may also decrease, leading to an increase in “biodiversity” over time. These are two balancing feedback loops with delays that might lead to oscillation in the system in relation to the achievement of the established objectives (i.e., the achievement of an attractive neighborhood and high‐quality BG public spaces). Both loops (and therefore both goals) are closely interconnected since they have three variables in common (“biodiversity,” “quality of BG public spaces,” and “use of BG public spaces”). In particular, the achievement of the objective “attractiveness of local area” may imply the non‐achievement of the objective “high‐quality BG public spaces.”

**Figure 7:**
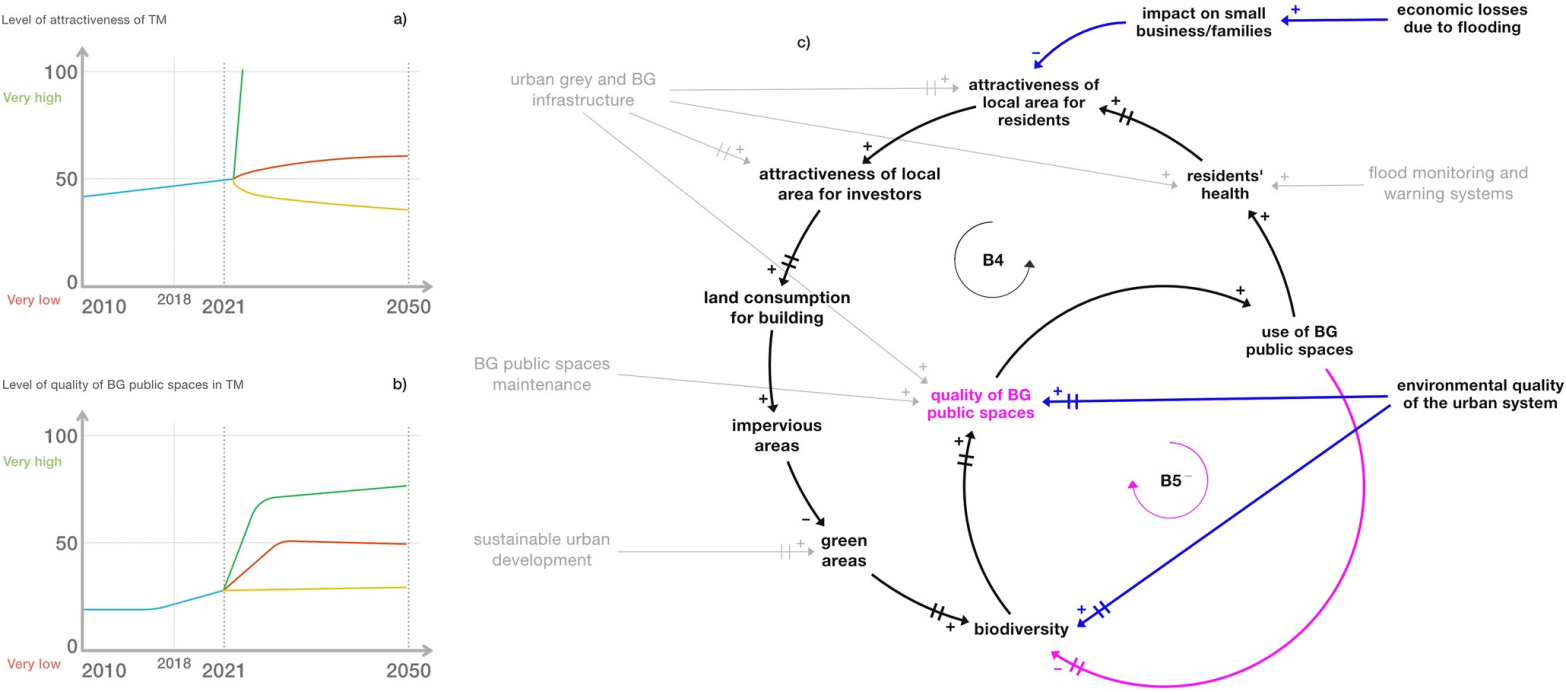
(a) BOT graph of the variable “attractiveness of local area” created by the stakeholders during a participatory workshop. (b) BOT graph of the variable “quality of Blue‐Green (BG) public spaces” created by the stakeholders during a participatory workshop. In both graphs the blue line represents the past behavior of the variable, the yellow, red, and green ones respectively the feared, most likely, and desired future. (c) Attractiveness of local area and quality of BG public spaces feedback loops (respectively B4 and B5). Loop B4 is the one in black, while loop B5 is the one in pink. The variables “biodiversity,” “quality of BG public spaces,” and “use of BG public spaces” are in common between the two loops. The variables in gray are measures/actions; simple causal relationships between variables outside the feedback loops (exogenous variables) are in blue. Changing times (delays) are represented by bars on the arrows.

In Figures 7a and 7b the feared futures (yellow lines) of the variables were represented by stakeholders with a low but (quite) constant trend due to both a lack of money for new investments and the effect of interventions first implemented in 2018.

To guarantee that the CLD correctly represents the constant trend of the feared futures it is necessary to consider simultaneously in Figure 7c: (a) the effect on the loops of external variables, such as “impact on small business/families” due to “economic losses” caused by flooding for loop B4 and “environmental quality of the urban system” for loop B5, and (b) the maintenance of pre‐existing measures. According to stakeholders, by activating flood risk mitigation/prevention measures, the variables “attractiveness of local area” and “quality of BG public spaces” may behave similarly to either the most likely futures (red lines) or the desired futures (green lines) in Figures 7a and 7b. The CLD can integrate this perception (see Figure 7c), provided that several actions, such as “sustainable urban development” and “urban gray and BG infrastructure,” are implemented simultaneously. Indeed, “sustainable urban development” might lead to an increase in “green areas” and “biodiversity” over time, continuing to guarantee, in the long run, the achievement of both objectives, while “urban gray and BG infrastructure” directly act on both variables. The difference between the desired and most likely futures depends on the degree to which the measures are activated. In particular, in the case of the desired futures, all the measures are applied and/or fully functioning and effective; while in the case of the most likely futures not all measures are applied, or they are not fully functioning and effective. Although the desired future of the variable “attractiveness of local area” suggests that very high levels of attractiveness may be achieved in a very short time (Figure 7a), it was specified by stakeholders that residents may not want the neighborhood to be too attractive because this would result in an exponential increase in housing prices. Therefore, ideally, the CLD narrative should represent a desired future in which the degree of attractiveness grows with time but not excessively. To this end, implementing measures that rapidly increase attractiveness could go against the wishes of residents.

## 5 Discussion

This section mainly discusses to what extent the present work provides an answer to the research questions raised in Section 1.

First, this work has demonstrated the importance of the integration between purely technical variables reflecting hydrological dynamics and non‐technical ones (such as social, economic, and environmental) in the analysis and management of flood risk within an expanding urban context. The urban system consists of several interacting elements and those belonging to the hydrological sub‐system represent only a part of the whole picture. This work has considered mechanisms and influences that have hitherto only been partially investigated. More specifically, it has shown that: (a) the dynamics that are activated in an urban system do not only concern the hydrological sub‐system, but also affect among the others the built environment, and the socio‐economic realm (e.g., influencing the “attractiveness of the local area and “residents' health”); (b) the stakeholders are mainly interested in understanding the implications related to exposure and vulnerability to risk, such as the “public realm damage” and “quality of BG public spaces.” This has a significant impacts for decision‐makers, as the identification of the most effective flood risk mitigation/prevention measures requires a deep understanding of the multi‐dimensional impacts and benefits they can have. For instance, the implementation of “urban gray and BG infrastructure” could positively impact hydrological variables, but also have an influence on other aspects such as “biodiversity,”“ attractiveness of local area” or “quality of BG public spaces.”

Regarding the second research question, the multi‐step process of knowledge gathering and structuring in the form of a CLD, provided a better understanding of flood risk in the urban context. The iterative integration of scientific and stakeholders' expert‐based knowledge allowed building the system structure of such complex topic considering a multiplicity of variables and processes (including also the social and environmental realms) that are often neglected. A set of semi‐structured interviews allowed integrating within the CLD several variables and highlighting critical interconnections among those, such as between the “urban drainage systems capacity” (Water management thematic cluster) and “environmental quality of urban systems” (Natural capital thematic cluster), providing useful system insights. Once the diagram integrated scientific and stakeholder knowledge, specific participatory exercises were used for its revision, validation and integration as well.

Lastly, CLDs are powerful tools for supporting the mapping and visualization of the interactions between the different system components, ultimately helping to describe the complex set of interconnections and feedback loops affecting its dynamic evolution. Exploring the system's structure through the use of CLDs may provide insights into behavioral trends (also affected by measures implementation), which is useful to support decision‐making processes at a planning/strategic level. Compared to more recent works adopting a similar approach to flood risk analysis (e.g., Dzulkarnain et al., 2019; Fenner et al., 2019), two main elements of innovation of the present work are related to (a) the detailed analysis of the feedback loops with focus on their impacts on key variables, and (b) the validation of the model causal structure and the construction of BOT graphs for key variables with stakeholders after CLD development. Specifically, the construction of BOT graphs expanded the potential of feedback loops in hypothesizing system behavior providing valuable support to decision‐makers in identifying, analyzing and prioritizing different flood risk mitigation/prevention actions.

Despite the scientific advancements associated with this work, a few issues are still open and need further research. First, the information provided by a qualitative model, although detailed and relevant, may not be enough to support the operationalization of an adaptive strategy. For this reason, the research activity is already being oriented toward the construction of a quantitative System Dynamics model, that is, a Stock and Flow (SF) model. Second, the integration of CLD narratives with BOT graphs has its own limitations, especially regarding models of high complexity. The isolation and examination of specific dynamics may produce results which are misrepresentative of the system functioning as a complex whole. The SF model may help overcome this limit. To handle the system complexity, the quantitative model can be organized into interconnected thematic sub‐models representing key processes and elements of an urban system exposed to flood risk, while providing a clear picture of the entire system. Third, despite the effectiveness of the proposed “expert‐based” modeling approach for the holistic analysis of the flood risk and urban dynamics, the modeling process should include the perspectives of other important interest groups that for now have not contributed to the model building. The current validated System Dynamics model can be used to simulate future scenarios by different group of stakeholders, and it could be further modified in the future, if needed. This may ensure that equity and equality aspects as well as justice procedures would be effectively incorporated into the participatory modeling process. In this direction, other strategies could be implemented. For instance, in the modeling process some of them were already carried out, such as using different engagement methods, encouraging knowledge exchange between stakeholders, employing skilled facilitators who can guide the participatory modeling process, etc. (see e.g., Ansell & Gash, 2007; Rittel & Webber, 1973).

## 6 Conclusions

Most of the existing modeling tools for supporting flood risk assessment and management in urban areas provide a limited understanding of multiplicity of dynamic interactions existing in a complex and evolving urban system. This also occur as a limited integration of stakeholders knowledge. The present work adopts System Thinking approaches and participatory System Dynamics modeling tools for explicitly including flood risk and flood risk mitigation in the analysis of urban development dynamics, ultimately providing an improved understanding of system state and evolution under different conditions. An improved conceptual modeling, based on Causal Loop Diagram allowed to: (a) integrate hydraulic aspects related to flood risk with other aspects (social, economic, and environmental) that are highly relevant to analyze urban dynamics (in the present work, with specific reference to a regeneration process); (b) explicitly integrate the flood phenomenon (and flood reduction measures) with the characteristics of the affected system, thus making preliminary assumptions on the behavior of key system variables under different conditions. The adopted methodology heavily relies on participatory modeling activities, and pursues the combination of scientific and stakeholder knowledge. Specific reference was made to the Thamesmead case study (London, UK). However, the developed modeling approach is suitable for replication in other projects focused on urban regeneration. Considering some potential limitations of this work, further research activities are already being oriented to the development of a quantitative Stock and Flow model and a larger stakeholder engagement.

## Supporting information

Supporting Information S1

## Data Availability

The model and data developed in the study are available at Zenodo (Coletta et al., 2023). Details on the interviews structures and involved stakeholders in this study are available in Supporting Information S1. PLE 8.1.2 of the Vensim® software used for developing the Causal Loop Diagrams is available for free for personal and educational use.
