# Supplementary material for: Participatory Causal Loop Diagrams Building for Supporting Decision‐Makers Integrating Flood Risk Management in an Urban Regeneration Process
Source: Earths Future. 2024 Jan 29;12(1):e2023EF003659. doi: 10.1029/2023EF003659 (PMC10909559; doi:10.1029/2023EF003659)
Supplement: Supplementary file 1 — Supporting Information S1 [file EFT2-12-e2023EF003659-s001.pdf]

Earth's Future

Supporting Information for

Participatory Causal Loop Diagrams Building for Supporting Decision-Makers Integrating Flood Risk Management in an Urban Regeneration Process

Virginia R. Coletta[1,2], Alessandro Pagano[2], Irene Pluchinotta[3], Nici Zimmermann[3], Michael Davies[3], Adrian Butler[4], Umberto Fratino[1], and Raffaele Giordano[2]

[1]Department of Civil, Environmental, Land, Construction and Chemistry, Polytechnic University of Bari, Bari, IT

[2]Water Research Institute - National Research Council, Bari, IT

[3]Institute for Environmental Design and Engineering, The Bartlett Faculty of the Built Environment, University College London, London, UK

[4]Department of Civil and Environmental Engineering, Imperial College London, London, UK

Contents of this file

Tables S1 to S6

Introduction

This section includes additional details related to (1) semi-structured interviews, (2) interviews/correspondences and engaged stakeholders, (3) Thamesmead flood Causal Loop Diagram (CLD) variables definitions, (4) the workshop agenda on the CLD causal structure validation and Behaviour Over Time (BOT) graphs construction, (5) stakeholders involved in the first workshop, (6) short description of the main feedback loops.

Table S1 Semi-structured interviews guideline on flood risk and past flooding events

Each question is associated with an objective. Depending on the answer to question 1, the interview proceeded differently (asking questions 2 to 10 if stakeholder answers yes or 11 and 12 if stakeholders answer no to question 1). Similarly, to the first question, the final questions (i.e., 13 and 14) are the same for all experts.

#	QUESTION	OBJECTIVE
1	Based on your own knowledge, have flooding events occurred in the area in the past?	Understand why it is important to investigate flooding in the area
	**YES**	
2	When?	Collect information on past flooding events
3	Do you think flooding may be a risk currently and/or in the future?<br>• If so, why? Are there transformations (e.g., urban transformations, climate change) taking place that may involve this?<br>• If not, why?	Understand why it is important to investigate flooding in the area
4	What kind of flood events were these (i.e., pluvial flooding, river flooding, groundwater flooding)?<br>What do you think were the causes? (e.g., drainage systems did not work, it rained for many days)	Understand what type of flooding the area is most susceptible to
5	Has there been any damage to the built environment (e.g., buildings and road, electrical, gas, and telecommunication infrastructure)?<br>• If so, what type of damage occurred (e.g., buildings filled with water, roads were inaccessible, electricity went off)?<br>Was there any damage to the basements or ground floors of buildings?<br>• If so, were the basements protected in any way?	Investigate damage to the built environment due to flooding
6	Have productive activities been affected? How?<br>Did flooding affect the price of dwellings? How?	Investigate damage to economy due to flooding
7	During the flood events, what was the role of the Sustainable Urban Drainage Systems (network lakes, canals)?<br>• Did they work properly? If not, why? Was it a design or management problem?	Investigate the performance of drainage systems in the area when the event took place
8	Were there warning and monitoring systems in place?<br>• If so, which ones (e.g., rainfall monitoring/forecasting, water level and velocity sensors in the river)?<br>Why did they not work?<br>Have you been alarmed in time by someone?	Investigate whether there were any warning and monitoring systems in the area and their effectiveness when the event took place

9	Have individual prevention measures been implemented?<br>• If so, what kind (e.g., leaving the ground floor of buildings vacant)?<br>• Why did they not work?<br>Have collective prevention measures been implemented?<br>• If so, what kind (e.g., ensure the functionality of drainage systems)?<br>• Why did they not work?	Investigate whether any prevention measures were implemented and their effectiveness when the event took place
10	What post-event intervention measures have been taken? Were they measures to restore the damaged system (e.g., rebuild buildings and infrastructure, improve drainage systems) or measures to prevent damage in the event of future flooding (e.g., activities to engage the community, insurance, and sustainable land use policies)?<br>• If so, who intervened? (e.g., municipality, national government)	Investigate what was done after the event and with which funds
	**NO**	
11	Do you think that no flood events have occurred in the past because the system is not exposed to risk (e.g., there is never heavy or long-lasting rainfall) or because risk mitigation measures (e.g., drainage systems) are effective?	Understand the susceptibility of the area to flooding and investigate the performance of mitigation measures in the area
12	Do you think that currently and/or in the future there may be flood risk in the area?<br>• If so, why? Are there transformations (e.g., urban transformations, climate change) taking place that may involve this thus changing the system's risk levels?<br>• If not, why?	Understand why it is important to investigate flooding
	FINAL QUESTIONS FOR EVERYONE	
13	(if appropriate) Is there anyone else you think we could usefully speak to?	Stakeholder snowballing
14	Thank you for your time. Is there anything else you would like to tell me about the topics we discussed today, on flood risk and flood events in the area?	Wrap up

Table S2 Details on interviews/correspondences and engaged stakeholders

INTERVIEW/ CORRESPONDENCE REFERENCE	INTERVIEW/ CORRESPONDENCE DATE	STAKEHOLDER ORGANIZATION	STAKEHOLDER ROLE
Int. 1	23/02/2021	Environmental Non-Governmental Organisation	Senior Manager
Int. 2	26/02/2021	Local Authority	Flood Risk and Development Manager
Int.3	31/03/2021	Housing Association/Developers	Director
Int.4	28/05/2021	Company of consulting and engineering/ architectural design	Director
Correspondence 1	16/02/2021	Housing Association/Developers	Sustainability Manager
Correspondence 2	23/02/2021	Environmental Non-Governmental Organisation	Senior Manager
Correspondence 3	1/03/2021	Local Authority	Flood Risk and Development Manager
Correspondence 4	23/03/2021	Local Authority	Project Officer

Table S3 Flood Causal Loop Diagram variables definitions

VARIABLE	DESCRIPTION
climate change	State of change in the climatic conditions over time that is identified as changes in the mean and/or the variability of its properties, that persists for an extended period
extreme tide levels intensity/frequency	Magnitude and occurrence rate per year of exceedance of tide threshold levels
extreme storm events intensity/frequency	Magnitude and occurrence rate per year of storm events extremes in the historical distribution
rainfall intensity/frequency	Magnitude and return period of precipitation events

soil retention capacity	Soil ability to storage water and make it sufficiently available for plant use
surface runoff	Precipitation runoff over the landscape
urban stormwater system capacity	The water volume that the sewage system can take without surcharging or flooding
existing SuDS (lakes and canals) capacity	Remaining volume of the networks of the existing Sustainable urban Drainage Systems (SuDS)
urban drainage systems capacity	The amount of water that can be stored by drainage systems (both ditches/dykes system and SuDS) without surcharging or flooding
groundwater level	The upper level of an underground surface in which the soil is permanently saturated with water
river water level	A measure of the depth of water in a river relative to an arbitrary point (e.g., the riverbed)
ditches and dykes system capacity	Available volume of ditches and dykes system
sediments and pollutants removal	Interception and filtration of sediments and pollutants present in the water and/or air
Thames tidal defences effectiveness	The capability of Thames tidal defences of producing the desired protection reducing, or ideally preventing, damage by flood water
sea water level	The level corresponding to the surface of the sea at mean level between high and low tide
flood	A temporary coverage with water of an area not normally covered by water
pluvial flood	Type of flooding that results from the lack of urban stormwater system capacity
groundwater flood	Type of flooding occurring when the natural water level below ground rises to well above what can be accommodated
tidal river flood	Type of flooding, usually very sudden, that results from the sea
fluvial flood	Type of flooding occurring when urban drainage systems no longer have capacity
environmental quality of the urban system	The combination of water, air, and soil quality, and the aesthetic and ecosystemic value of the area
green areas	All urban land covered by vegetation of any kind
impervious areas	Surfaces, completely human-created, that allow little or no stormwater infiltration into the ground
housing demand	A market driven concept that relates to the type and number of houses that households will

	choose to occupy based on preference and ability to pay
population growth	The increase in the number of individuals in the population
infrastructure damage	All detrimental effects on basic structures and facilities (highways, electrical, gas, and telecommunication) provoked by flooding
public realm damage	All detrimental effects on all parts of the built environment where the public has free access (streets, squares, parks, open spaces, waterfronts, and public transit systems) provoked by flooding
private properties damage	All detrimental effects provoked by flooding on the interiors and structures of properties owned by private parties
private properties value	The worth of a piece of real estate based on the price that a buyer and seller agree upon, determined by what the market bears
economic losses	The value of direct (e.g., cost of repairs) and indirect (e.g., lost income losses) financial losses due to flooding
productive activities operation	The functioning of activities that have economic value in the marketplace
attractiveness of local area for residents	The feature that makes the area appealing to residents and meets their needs
attractiveness of local area for investors	The quality of the area that make it interesting to investors
impact on large businesses	Financial effect on large businesses
impact on small businesses/families	Financial effect on small businesses and families
insurance policies	Insurance tools that limit the impacts of hazards on insured people, objects, or organizations through the transfer of these impacts to an insurer who will provide for economic compensation
individual prevention measures	Self-insurance initiatives against flooding
residents' awareness and preparedness to flood event	The extent of common knowledge about flooding risk and the actions that can be taken to reduce exposure and vulnerability to it, as well as capabilities and actions of community to effectively anticipate, respond to, and recover from, the impacts of flooding events
quality of BG public spaces	Distinctive attribute of the benefits blue and green (BG) public spaces provide, which might be

	important determinants for how and how frequently people use them and for human well-being
biodiversity	Coexistence, in the same ecosystem, of a variability of living organisms from all sources, within species and between species
use of BG public spaces	Attendance degree of BG public spaces by people
residents' health	State of citizens' physical and mental well-being
land consumption for building	The conversion of land with healthy soil and intact habitats into areas for urban human settlements
water demand	The volume of water requested by users to satisfy their needs
urban grey and BG infrastructure (permeable pavements, attenuation tank, green roofs, swales, retention areas) implementation and maintenance	Development and functional preservation of grey and green measures
existing systems maintenance	The art of keeping existing systems in condition to fulfil adequately the purposes for which they were intended
BG public spaces maintenance	Set of activities necessary to keep blue and green public spaces in good condition and in full working order
sustainable urban development	The persecution of urban form that synthesises land development and nature preservation and places the protection of natural systems into a state of vital equipoise
flood risk monitoring and warning systems	Tools and systems supporting operate during the flooding event
local community engagement	The process of working collaboratively with and through groups of residents affiliated by interest or similar situations to address issues affecting their well-being

Table S4 Workshop agenda of Thamesmead CLD causal structure validation and BOT graphs construction with stakeholders

Date: 9/09/2021
Time: 2h
Location: online, using Microsoft Teams
Aims

- To present the flood CLD
- To validate the general structure and specific elements
- To build the BOT of some key variables

TIME	ACTIVITY	OBJECTIVE
10 min	Welcome and introduction	Warm up for orientation and goal clarification
3 min	Flood CLD presentation	Presentation of the modelling process, prepare the participants for the next activities
47 min	Flood CLD validation (semi-structured interviews style)	To reach consensus over the model structure
5 min	BOT activities presentation	Activity clarification
30 min	BOT graphs construction (5 variables)	-
10 min	BOT presentation	To share BOT graphs created by each group
10 min	Evaluation<br>Next steps and closing	-

Table S5 List of the stakeholders involved in the first workshop

STAKEHOLDER	ORGANIZATION	ROLE
Stakeholder 1	Housing Association/Developers	Director
Stakeholder 2	Housing Association/Developers	Head of Landscape & Placemaking
Stakeholder 3	Local Authority	Flood Risk and Development Manager
Stakeholder 4	Environmental Non-Governmental Organisation	Senior Manager
Stakeholder 5	Company of consulting and engineering/ architectural design	Director
Stakeholder 6	Local Authority	Project Manager
Stakeholder 7	Local Authority	Manager of Operations

Table S6 Short description of the main feedback loops

LOOP	DYNAMICS	INTERNAL VARIABLES	BEHAVIOUR MODE
B1	infrastructure damage	• residents' health<br>• attractiveness of local area for residents<br>• attractiveness of local area for investors<br>• land consumption for building<br>• impervious areas<br>• green areas<br>• soil retention capacity<br>• surface runoff<br>• urban stormwater system capacity<br>• pluvial flood<br>• flood<br>• infrastructure damage	oscillation
B2	public realm damage	• economic losses<br>• impact on small business/families<br>• attractiveness of local area for residents<br>• housing demand<br>• land consumption for building<br>• impervious areas<br>• green areas<br>• soil retention capacity<br>• surface runoff<br>• urban stormwater system capacity<br>• pluvial flood<br>• flood<br>• public realm damage	oscillation
B3	private properties damage	• economic losses<br>• impact on small business/families<br>• residents' awareness and preparedness to flood event<br>• individual prevention measures<br>• private properties damage	oscillation
B4	attractiveness of local area	• land consumption for building<br>• impervious areas	oscillation

		• green areas<br>• biodiversity<br>• quality of BG public spaces<br>• use of BG public spaces<br>• residents’ health<br>• attractiveness of local area for residents’<br>• attractiveness of local area for investors	
B5	quality of BG public spaces	• use of BG public spaces<br>• biodiversity<br>• quality of BG public spaces	oscillation
R	residents’ health	• attractiveness of local area for residents<br>• use of BG public spaces<br>• residents’ health	exponential growth/decline
